# Identification of Bioactive Peptides from *Nannochloropsis oculata* Using a Combination of Enzymatic Treatment, in Silico Analysis and Chemical Synthesis

**DOI:** 10.3390/biom12121806

**Published:** 2022-12-02

**Authors:** Maria Hayes, Leticia Mora, Simona Lucakova

**Affiliations:** 1 Food BioSciences Department, Teagasc Food Research Centre, Ashtown, D15 Dublin, Ireland; 2 Instituto de Agroquímica y Tecnología de Alimentos, Burjassot CSIC, 46980 Valencia, Spain; 3 Institute of Chemical Process Fundamentals of the Czech Academy of Sciences, Rozvojova 135/1, 165 02 Prague, Czech Republic

**Keywords:** microalgae, protein hydrolysates, pro-peptides, Angiotensin Converting Enzyme-1 (ACE-1), in silico analysis, *Nannochloropsis oculata*, health, functional foods, metabolic syndrome

## Abstract

In vitro ACE-1 inhibitory peptides were characterised previously from a number of microalgal species including *Spirulina platensis* (peptide IAPG), *Chlorella vulgaris* (peptides FDL, AFL, VVPPA), *Isochrysis galbana* (peptide YMGLDLK), *Chlorella sorokiniana* (peptides IW and LW) and indeed *Nannochloropsis oculata* (peptides GMNNLTP and LEQ). The isolation of protein from *Nannochloropsis oculata* using a combination of ammonium salt precipitation and xylanase treatment of resulting biomass combined with molecular weight cut off filtration to produce a permeate and characterisation of bioactive peptides is described. The Angiotensin-1-converting enzyme (ACE-1) IC_50_ value for the generated permeate fraction was 370 µg/mL. Ninety-five peptide sequences within the permeate fraction were determined using mass spectrometry and eight peptides were selected for chemical synthesis based on in silico analysis. Synthesized peptides were novel based on a search of the literature and relevant databases. In silico, simulated gastrointestinal digestion identified further peptides with bioactivities including ACE-1 inhibitory peptides and peptides with antithrombotic and calcium/calmodulin-dependent kinase II (CAMKII) inhibition. This work highlights the potential of *Nannochloropsis oculata* biomass as both a protein and bioactive peptide resource, which could be harnessed for use in the development of functional foods and feeds.

## 1. Introduction

Microalgae are a diverse group of photosynthetic, microscopic organisms including eukaryotic unicellular microalgae and prokaryotic cyanobacteria [1]. Considered within the EU as a potentially viable source of alternative proteins, due to their ease of cultivation and high protein content, they also can transform solar energy into organic chemical energy and are highly efficient at fixing carbon dioxide (CO_2_) [2]. Microalgae are a renewable and sustainable biomass for high-value, bioactive ingredient generation for use in pharmaceuticals, food ingredients or cosmetics and grow in either raceway pond systems or different bioreactors for controlled production [3]. *Nannochloropsis* species are considered as one of the most promising strains for cultivation in large scale systems due to their rapid growth rate and accumulation of significant amounts of lipids, eicosapentaenoic acid [4] and proteins [5,6]. To date, several studies identified commercially attractive bioactive compounds from *Nannochloropsis* sp. biomass [7] and have observed the potential positive health benefits of *Nannochloropsis* sp. biomass or isolated bioactive peptides on hypertension [6,8,9] blood glucose levels and cholesterol [10,11,12].

Hypertension is a serious medical condition that significantly increases the risk of heart, brain, kidney and other diseases associated with metabolic syndrome (MS). According to the WHO, three types of drugs are successful for treatment of hypertension: Thiazide and Thiazide-like agents, Angiotensin-1-converting enzyme (ACE-1; EC 3.4.15.1) inhibitors and long-acting Dihydropyridine calcium channel blockers [13]. ACE-1 plays a crucial role in the regulation of blood pressure and salt water balance within mammalian Renin-Angiotensin-Aldosterone Systems (RAAS) and Kinin-Kallikrein systems [14]. Moreover, use of ACE-1 inhibitors and angiotensin receptor blockers may affect COVID-19 through modulating levels of Angiotensin-converting enzyme 2 (ACE-2; EC 3.4.17.23), the cell entry receptor for SARS-CoV2 [15]. However, commercially available ACE-1 inhibitory drugs including Benazepril (sold under commercial name Lotensin^®^), Captopril©, Enalapril (commercially sold as Vasotec^®^) and Fosinopril (Monopril^TM^, trade mark of E.R. Squibb and sons) may have side effects for users including the development of dry cough, increased potassium levels in the blood, dizziness or headaches. Alternatives to these synthetic drugs include ACE-1 inhibitory peptides derived from protein food sources including dairy [16], meat [17] and fish [18]. ACE-1 inhibitory peptides are effectively used to maintain normal blood pressure and prevent hypertension and are found as the bioactive components of protein hydrolysates or concentrates sold for prevention of high blood pressure [17]. Food-derived ACE-1 inhibitory peptide concentrates are in several commercially available functional food products and supplements including PreCardix^®^ (produced by Norwegian company Marealis which, received approval for use as a novel food in Europe from EFSA in 2018) derived from shrimp (*Pandalus borealis*) and Calpis© containing the tripeptides IPP and VPP and derived from fermented dairy proteins [19].

Limited studies exist concerning the use of *Nannochloropsis* sp. as a source of protein, protein hydrolysates or bioactive peptides. Recently, Verspreet et al. (Verspreet et al., 2021) demonstrated the ACE-1 inhibitory activity of whole, disrupted *Nannochloropsis gaditana* which inhibited ACE-1 by 87.1% when assayed at a concentration of 1 mg/mL compared to the control Captopril© assayed at a concentration of 0.5 mg/mL [6]. Enzymatic treatment of biomass is a useful strategy for generation of bioactive peptide containing concentrates. It can enhance protein release from biomass [20] and increase the bioavailability of proteins, peptides and amino acids [21]. In addition, it may be used to enhance the palatability of proteins depending on enzyme selection. Moreover, enzyme treatment of biomass may reduce the potential of a protein source to cause allergy. Previously, Alcalase treated *Nannochloropsis oculata* was found to inhibit ACE-1 by 77.6% when assayed at a concentration of 1 mg/mL and had an IC_50_ value of 0.126 mg/mL. The ACE-1 inhibitory penta-peptide with the amino acid sequence Leu-Val-Thr-Val-Met and a molecular weight of 561 Da was characterised from this hydrolysate. The ACE-1 IC_50_ value of this purified peptide was determined as 18 µM [9]. Moreover, pepsin treated *Nannochloropsis oculata* generated the ACE-1 inhibitory peptides with amino acid sequences Gly-Met-Asn-Asn-Leu-Thr-Pro (MW 728 Da) and Leu-Glu-Gln (MW 369 Da) with characterised ACE-1 IC_50_ value of 123 and 173 µM, respectively [8].

In this study, protein was extracted from *Nannochloropsis oculata* biomass using ammonium sulphate precipitation and the resulting protein containing fraction was treated with the enzyme xylanase (Merck, Dublin, Ireland) and permeates generated using molecular weight cut off (MWCO) filtration. The permeate fraction was assessed for its ability to inhibit the enzyme ACE-1 and peptide sequences were determined using mass spectrometry. Eight peptides were selected for chemical synthesis based on in silico analysis of their amino acid sequences. All peptides identified were novel and were found to inhibit ACE-1 in vitro at physiologically relevant concentrations, relevant to the positive control Captopril©. Selected peptides were also confirmed to have antibacterial activity and cyclooxygenase 1 and 2 inhibitory activities.

## 2. Materials and Methods

**Figure 1:**
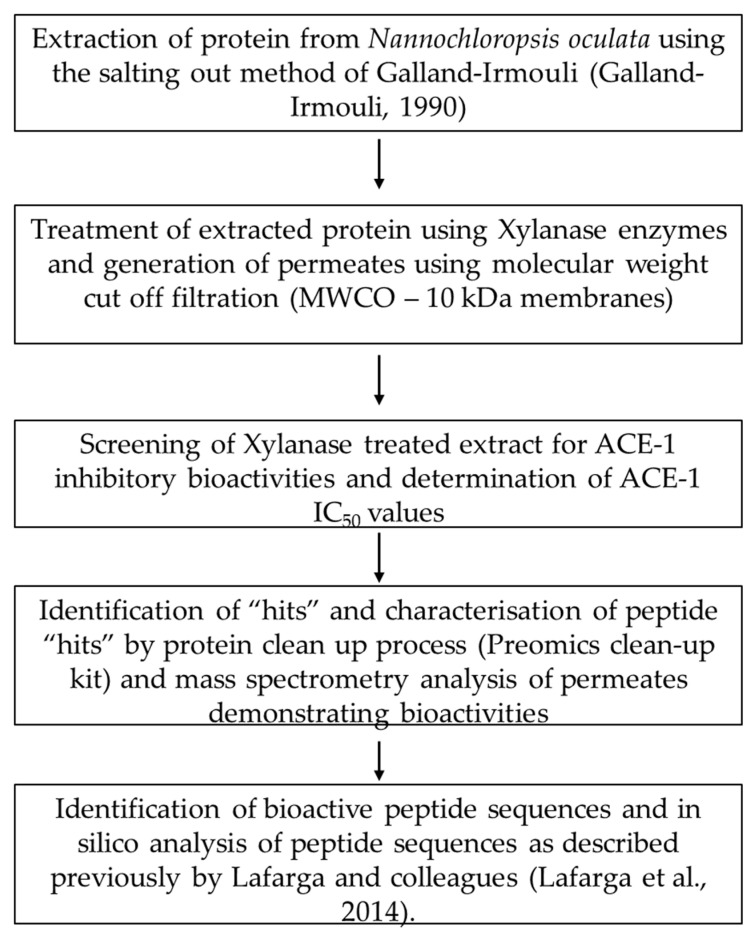
Workflow diagram of processes applied to generate bioactives from *Nannochloropsis oculata* [22,23].

The workflow is outlined in Figure 1.

### 2.1. Materials

Xylanase enzyme from *Aspergillus oryzae* (>2500 units/g) was supplied by Merck (Dublin, Ireland). Tebu Bio (TebuBio Ltd., Peterborough, UK) supplied the ACE-WST inhibitor screening assay kit. The positive ACE-1 inhibitor control, Captopril© was purchased from Merck (Merck, Dublin, Ireland). All other chemicals used were of analytical grade. *Nannochloropsis* sp. biomass was supplied freeze-dried by VITO (Mol, Belgium) as part of the IDEA project. *Escherichia coli* DSMZ 101 was purchased from DSMZ (DSMZ, Germany). Media and broth used to culture this pathogenic strain of bacteria was LB broth.

### 2.2. Protein Extraction from Supplied Nannochloropsis sp. Biomass

Crude protein was extracted from *Nannochloropsis* sp. using the method previously described by Galland-Irmouli et al. (1999) [22] and Fitzgerald et al. [24] previously. Fitzgerald and colleagues used this method followed by hydrolysis of the resulting protein with proteolytic enzymes to generate bioactive peptides, specifically Renin inhibitory peptides from the red macroalga *Palmaria palmata* (Irish Dulse) [24]. *Nannochloropsis* sp. was supplied by VITO, Mol, Belgium as part of the IDEA Interreg NEW project. Briefly, 10 g of dried *Nannochloropsis* sp. was suspended in 1 L of ultrapure water. After ultrasonication for 1 h, the microalgal solution was left to stir overnight on a magnetic stirrer plate (C-MAGHS 7KAMAG, IKA-Werke GmbH & Co. KG, Staufen, Germany) at 4 °C. The solution was then centrifuged at 10,000× *g* for 1 h and the supernatant decanted. The pellet fraction was suspended in 200 mL of ultrapure water and subjected to a second extraction procedure as described above. Both supernatants were pooled together and subsequently brought to 80% ammonium sulfate saturation, stirred for an hour at 4 °C, and centrifuged at 20,000× *g* for 1 h to precipitate the protein fraction. The precipitates were subsequently dialyzed using 3.5 KDa MWCO dialysis tubing (Fisher Scientific, Waltham, MA, USA) against ultrapure water at 4 °C overnight. The precipitates were subsequently freeze-dried and stored at −80 °C until further use. The concentration of the crude *Nannochloropsis* sp. protein was quantified using an AOAC method. Extractions were performed in triplicate (*n* = 3).

### 2.3. Xylanase Treatment and Preparation of Molecular Weight Cut Off Permeate Fractions

Freeze-dried *Nannochloropsis* sp. protein biomass was resuspended in ddH_2_O (0.5 g protein extract: 100 mL ddH_2_O in triplicate) and hydrolysed using the xylanase enzyme. The sample solution was pre-incubated at 97 °C for 15 min to heat deactivate endogenous enzymes. The pH of the suspension was adjusted from 5.1 to 7.0 using 0.5 M NaOH. Subsequently, xylanase enzyme was added to the mixture at an enzyme: substrate ratio of 1:100 (*w*/*w*) to initiate the hydrolysis.

During hydrolysis, the suspension was incubated in a thermostatic shaking incubator (Cole Parmer Instrument Co. Ltd., Eaton Socon, UK) at 70 °C for 3 h with stirring at 200 rpm. After hydrolysis, xylanase was heat-deactivated by incubation of the suspension in a water-bath at 97 °C for 15 min. Following cooling to room temperature, the mixture was filtered through a 10-kDa molecular weight cut off (MWCO) filter using the Millipore lab-scale Tangible Flow Filtration (TFF) system (Millipore, Cork, Ireland). The membrane was cleaned using 0.1 M NaOH at 45 °C followed by washing with ddH_2_O prior to passing the hydrolysates through the system to generate 10-kDa permeate and retentate fractions.

### 2.4. Screening and Identification of ACE-1 Inhibitory Activity

The ACE-I inhibitory activity was determined using a 96 well plate bioassay supplied by Tebu-Bio (TebuBio Ltd., Peterborough, UK) in accordance with the manufacturers’ instructions. Briefly, the enzyme working solution and indicator working solution were prepared according to the manufacturer’s instructions. 20 µL of a 1% (*w*/*v*) solution of each permeate fraction and 46.02 mM of Captopril © (an ACE-1 inhibitory drug and positive control) dissolved in distilled water were added to the experimental sample wells of a 96-well microplate. Blanks were included. 20 µL of the substrate buffer was added to each well, followed by 20 µL of the enzyme working solution to experimental wells and blank one wells. The plate was incubated at 37 °C for 1 h. After incubation, 200 µL indicator working solution was added to each well, and the plate was incubated at room temperature for 10 min. Absorbance was subsequently measured at 450 nm on a microplate autoreader, and ACE-1 inhibitory activity was calculated using the following equation:

Percentage ACE-1 inhibition = (Absorbance of blank_1_ − Absorbance of sample)/(Absorbance of blank_1_ − Absorbance of blank_2_) × 100.

where; blank_1_ is the control without the addition of any inhibitor, blank_2_ is the reagent blank, and the inhibitor is the positive control (Captopril©) or test sample (protein hydrolysate fraction). Absorbance was measured using a plate reader at 450 nm. For IC_50_ determination, the ACE kit-WST was also used.

### 2.5. Preparation, Mass Spectrometry Analysis and Identification of Peptides

The microalgal permeate fraction was processed for mass spectrometry analysis using the Preomics Phoenix Cleanup Kit (96×), (Preomics, D-82152 Planegg/Martinsried, Germany) in accordance with the manufacturers’ instructions and described previously [25]. The sample was acidified and hydrophobic and hydrophilic contaminants removed using a series of wash steps and peptides eluted from the cartridge and prepared in loading buffer for LC-MS analysis. Peptides were identified using a mass spectrometer nanoESI qQTOF (6600 plus TripleTOF, AB SCIEX, Framingham, MA, USA) using liquid chromatography and tandem mass spectrometry (LC–MS/MS). A total of 1 μL of microalgal permeate was loaded onto a trap column (3 µ C18-CL 120 Ᾰ, 350 μM × 0.5 mm; Eksigent, Redwood City, CA, USA) and desalted with 0.1% TFA (trifluoroacetic acid) at 5 µL/min during 5 min. The peptides were then loaded onto an analytical column (3 µ C18-CL 120 Ᾰ, 0.075 × 150 mm) equilibrated in 5% acetonitrile 0.1% FA (formic acid). Elution was carried out with a linear gradient from 7 to 45% B in A for 20 min, where solvent A was 0.1% FA and solvent B was ACN (acetonitrile) with 0.1% FA) at a flow rate of 300 nL/min. The sample was ionized in an electrospray source Optiflow < 1 μL Nano applying 3.0 kV to the spray emitter at 200 °C. Analysis was carried out in a data-dependent mode. Survey MS1 scans were acquired from 350 to 1400 *m*/*z* for 250 ms. The quadrupole resolution was set to ‘LOW’ for MS2 experiments, which were acquired from 100 to 1500 *m*/*z* for 25 ms in ‘high sensitivity’ mode. The following switch criteria were used: charge: 1+ to 4+; minimum intensity; 100 counts per second (cps). Up to 50 ions were selected for fragmentation after each survey scan. Dynamic exclusion was set to 15 s. The system sensitivity was controlled by analyzing 500 ng of K562 protein extract digest (SCIEX); in these conditions, 2260 proteins were identified (FDR < 1%) in a 45 min gradient.

Protein Pilot v 5.0. (SCIEX) default parameters were used to generate peak list directly from 6600 plus TripleTOF wiff files. The Paragon algorithm of ProteinPilot v 5.0 was used to search different databases. Peptides were identified with a confidence of ≥95%.

### 2.6. In Silico Analysis

Peptides identified were compared to other bioactive peptides previously reported in the literature and to peptides found in BIOPEP-UWM http://www.uwm.edu.pl/biochemia/index.php/en/biopep [26] (accessed on 25 May 2022).

#### 2.6.1. Simulated Gastrointestinal (GI) Digestion Using Peptide Cutter

In silico analysis was used to determine potential survival of identified ACE-I inhibitory peptides in the GI tract. The four identified peptide sequences were assessed for potential cleavage by GI tract enzymes as described previously [24,27,28] using the software program Expasy PeptideCutter http://ca.expasy.org/cgi-bin/peptidecutter/peptidecutter.pl-accessed on the 25 July 2022. Simulated cleavage of the peptides with pepsin (pH > 1.3 and pH 2.0) (EC 3.4.23.1) and trypsin (EC 3.4.21.4) was carried out. Resulting peptide fragments were subsequently assessed for potential bioactivities and novelty in BIOPEP-UWM.

#### 2.6.2. Prediction of Peptide and Peptide Fragment Bioactivities

The potential bioactivities of peptides and peptide fragments identified and generated following simulated GI digestion were predicted using PeptideRanker [26] http://bioware.ucd.ie/~compass/biowareweb/-accessed on the 25 July 2022 and peptide scores calculated.

### 2.7. Chemical Synthesis, Primary Structure Determination and Theoretical Value Calculation of Peptide Sequences

Peptides identified as having potential bioactivities with the amino acid sequences NKFPYTTQ, VYNKFPYTTQ, LVGADAHALGVICS, VVGAVGAADLL, AGDVGFDPLGF, GDVGLF, YANDLLCMPI and KGGGSSAMGGRL were chemically synthesised by GenScript Biotech (Leiden, The Netherlands). GenScript also verified the purity of the peptide by analytical RP-HPLC–MS. The primary structure and the theoretical values of the selected peptides was determined using PepDraw (https://www2.tulane.edu/~biochem/WW/PepDraw/-accessed on 29 August 2022).

### 2.8. Cyclooxygenase Inhibition

In vitro assays to determine COX inhibition were performed using cell free assays as described previously [29]. The synthesised peptides were incubated independently with ovine recombinant COX-1 (Cayman Chemicals, Hamburg, Germany) or human recombinant COX-2 (Cayman Chemicals, Hamburg, Germany). The assay was carried out in accordance with the manufacturer’s instructions. Resveratrol (Merck, Dublin, Ireland) was used as a positive control.

### 2.9. Antimicrobial Screening

The antibacterial activity of synthesised peptides was determined using an overnight culture of the pathogenic bacteria in a modified agar well diffusion assay as described previously [30]. A zone of inhibition around wells containing the peptides indicates antibacterial activity. *Escherichia coli* DSM 301 and *Bacillus cereus* DSM 31 were used as indicator pathogen strains. Ampicillin (Merck, Dublin, Ireland) and Cecropin P1 (Merck, Dublin Ireland) were used as positive control antibiotics and antimicrobial peptides. Positive controls or test peptides were incubated (concentration 1 mg/mL) in a well (30 µL capacity) on the agar plate which was seeded with the pathogenic strain at an overnight culture to media ratio of 100 µL: 500 mL (*v*:*v*). Plates were left at 4 °C for 4 h and subsequently incubated at the optimum culture temperature and conditions for the pathogen in question. After 24 h, zones of inhibition indicated the presence of antibacterial activity.

## 3. Results

### 3.1. Extraction Yields for Protein and Xylanase Treated Permeate Fractions

**Figure 2:**
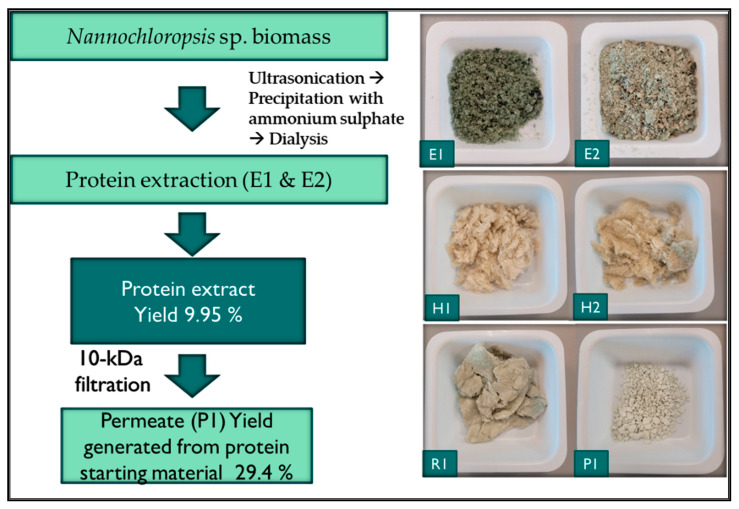
*Nannochloropsis* sp. protein treated fraction and permeate generation process and resultant protein extracts (**E1** & **E2**), proteins treated enzyme fractions generated using xylanase (**H1** & **H2**) and permeate (**P1**) and retentate (**R1**) fractions generated.

Methods to extract proteins from seaweeds developed to date are largely based on the methods of Fleurence [31] and Galland-Irmouli and Fleurence [22]. These methods involve the extraction of seaweed using deionized water and the generation of a supernatant and pellet (the pellet consisting of the non-soluble biomass). Protein is extracted by re-suspending the pellet in an alkaline solution (normally ammonium sulphate) and further separation of the supernatant and pellet, and subsequently combining the two supernatants as one final extract that contains the protein. However, there are several reports of protein yield variabilities with this method with some authors suggesting that preservation methods for seaweeds also have an impact on protein yields [32]. In this work, we used a combination of the Galland-Irmouli method [22] and xylanase treatment to add value to the resulting protein extract. The percentage yield of protein generated following dialysis and ammonium sulphate precipitation of protein was 9.95% (±1.95), (*n* = 2). The yield of 10 kDa permeate generated following MWCO filtration of the *Nannochloropsis* sp. protein treated with the enzyme xylanase was 29.4% of the starting whole microalgal biomass. The permeate fraction recovered (Figure 2) was used for bioassay work and was further characterised using mass spectrometry to identify peptide sequences following ACE-1 inhibitor screening. Following enzyme treatment and filtration of biomass, the colour changed from a green colour for the protein extract to an off-white colour in the end permeate produced.

### 3.2. Proximate Composition of Whole Alga and Protein Extracts

The protein content of whole *Nannochloropsis* sp. biomass and protein extracted from *Nannochloropsis* sp. was determined using a LECO FP628 (LECO Corp., Benton Harbor, MI, USA) protein analyzer and the Dumas method, according to AOAC method 992.15, 1995 [33]. The ash, moisture and fat contents were also determined and compared to whole *Nannochloropsis* sp. algae and extracts (Table 1). The protein content of *Nannochloropsis* sp. protein was 26.16% compared to a protein content of 27.95% in the whole microalga. The ash content of the generated protein extract was 27.78% compared to an ash content of 23.18 for the whole microalga. The lipid content of *Nannochloropsis* sp. starting biomass was significantly lower than that observed for other *Nannochloropsis* sp. (Table 1). This is because the starting biomass used in this study was defatted prior to use (VITO, Mol, Belgium). The protein content of the biomass used in this study was comparable to *N. gaditana* examined previously by Nacer and colleagues [10].

### 3.3. ACE-1 Inhibition and Characterisation of Bioactive Peptides Using Mass Spectrometry (MS)

The ACE-1 inhibitory activity of the 3-kDa permeate fraction generated from *Nannochloropsis* sp. protein treated with xylanase was determined using the ACE-1 inhibition colorimetric method as described previously [38]. The permeate fraction inhibited ACE-1 by 96.6% when assayed at a concentration of 1 mg/mL compared to the positive control Captopril^®^ which inhibited ACE-1 by 99.3% when tested at a concentration of 0.5 mg/mL. The IC_50_ value for the permeate fraction was determined as 0.37 mg/mL (370 µg/mL). This value compares favourably with previously identified ACE-1 inhibitor peptides identified from *Nannochloropis* sp. For example, an Alcalase^®^ protein hydrolysate generated from *Nannochloropsis oculata* previously, inhibited ACE-1 by 77.6% when assayed at a concentration of 1 mg/mL and had an IC_50_ value of 0.126 mg/mL (126 µg/mL). Samarakoon and colleagues previously identified two ACE-1 inhibitory peptides from a protein hydrolysate of *Nannochloropsis oculata* with the amino acid sequences GMNNLTP and LEQ. These were identified as having ACE-1 IC_50_ values of 123 µM and 173 µM, respectively [35].

Following MS analysis, a total of 95 peptides were identified in the *Nannochloropsis* sp. protein xylanase permeate fraction. However, only eight peptide sequences were synthesised based on in silico analysis. These peptides were AGDVGFDPLGF corresponding to a peptide sequence from a Chlorophyll a-b binding protein with a PeptideRanker score of 0.89 and other peptides including NKFPYTTQ corresponding to a peptide sequence from a phycocyanin protein (accession number: tr|S5FXR4|S5FXR4_9CYAN) and the peptide VYNKFPYTTQ with homology to a peptide sequence in the same protein. From a search for these peptides in UniProt (https://www.uniprot.org/uniprotkb/P27288/entry accessed on 25 July 2022) both peptides also correspond to f(61–68) and f(59–68) of protein R-phycocyanin alpha subunit Synechococcus sp. (accession numer: P27288 PHCA_SYNPW). Two other peptides including LVGADAHALGVICS corresponding to a peptide sequence found in a transcriptor initiation protein from Porphyridium sp. (accession number tr|A0A5J4YUD5|A0A5J4YUD5_PORPP) and the peptide VVGAVGAADLL corresponding to a peptide sequence found in the HEAT repeat-containing protein 1 OS = Ectocarpus siliculosus OX = 2880 GN = Esi_0125_0072 PE = 3 SV = 1 (accession number: tr|D7FJ23|D7FJ23_ECTSI) were also identified. Peptides were selected for synthesis based on their Peptide Ranker scores but also based on the presence of amino acids such as Q at the terminal end of the peptide.

### 3.4. In Silico Assessment of Identified Peptide Novelity and Additional Bioactivities

Following identification of the amino acid peptide sequences using MS, in silico methods were applied to these peptides to determine the novelty of the peptides and to predict further bioactivities that may be associated with them. Following assessment of the peptides using PeptideRanker (http://distilldeep.ucd.ie/~gianluca/cgi-bin/distill/predict_peptideranker) [39], AGDVGFDPLGF peptide was identified as having the greatest potential to be bioactive (0.89). A search in BIOPEP-UWM™ database of bioactive peptides and PepBank (https://biochemia.uwm.edu.pl/biopep-uwm/ and http://pepbank.mgh.harvard.edu; accessed on 2 August 2022) [26] found that all of the identified peptide sequences were novel. Fractions of these peptides generated following in silico gastrointestinal digestion Table 2) also predict that identified peptides could have antioxidant and Calcium/calmodulin-dependent protein kinases (CaMKs) inhibitory activities (CAMKII inhibitors). CAMKII inhibitors are key regulators of calcium signaling in health and disease and CaMKII inhibition displays cardioprotection [40].

### 3.5. Chemical Synthesis and Confirmation of Bioactivities In Vitro

**Figure 3:**
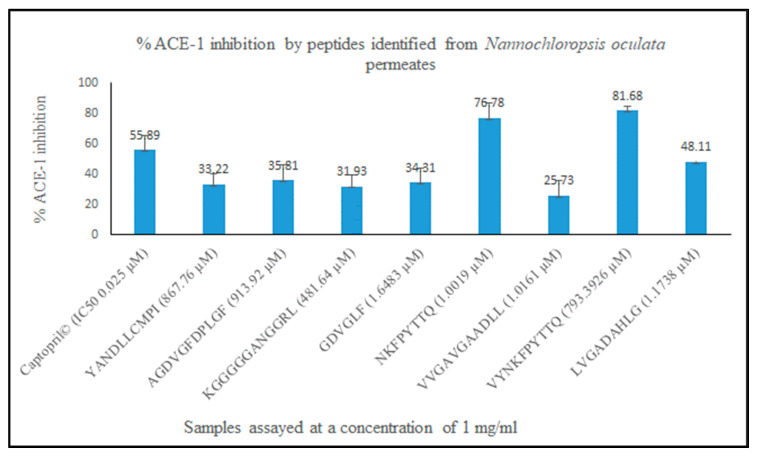
ACE-1 inhibitory activities of identified and synthesised peptides from *Nannochloropsis oculata* treated with xylanase.

**Figure 4:**
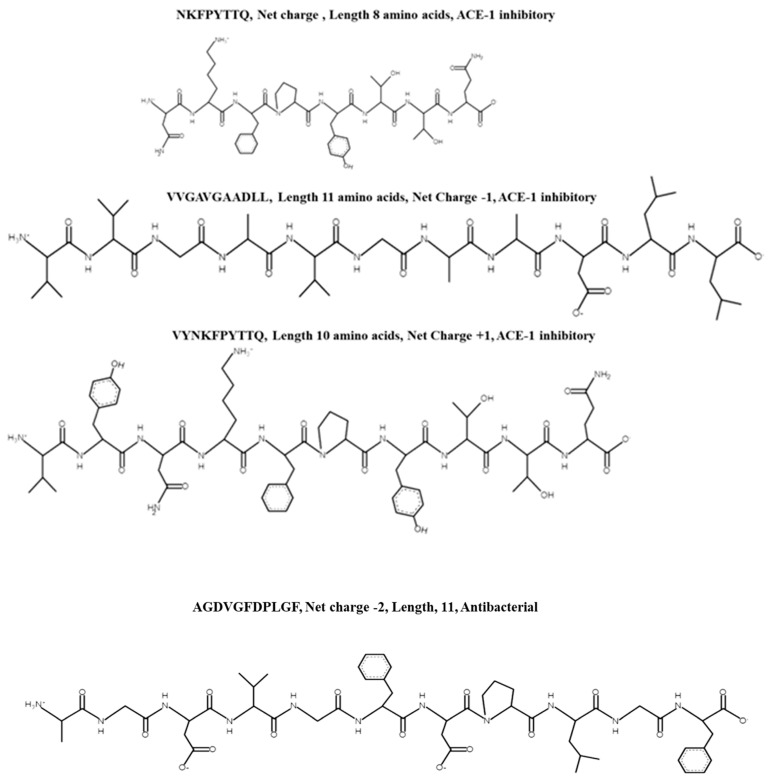
Chemical structure and characteristics of synthesised peptides from Nannochloropsis oculata xy-lanase treated protein with ACE-1 inhibitory activities. Chemical structures were drawn using PepDraw (PepDraw (tulane.edu))–accessed on 22 August 2022.

Eight peptides (shown in column 2 of Table 2) were chemically synthesised (Genscript Biotech, Leiden, The Netherlands) and subsequently used in ACE-1 and Cyclooxygenase (COX-1 and COX-2) inhibition and antibacterial bioassays to confirm bioactivities. These peptides were selected based on their Peptide Ranker scores and amino acid sequences. The ACE-1 and COX-1/COX-2 assays were performed as described previously described using commercially available kits from Cambridge BioSciences (Cambridge BioSciences, Cambridge, UK). The well diffusion bioassay was used to determine antibacterial activity of the peptides against pathogenic strains *Escherichia coli* and *Bacillus cereus* species (DSMZ, Braunschweig, Germany). Pathogens were selected based on in house availability. Only one peptide, peptide AGDVGFDPLGF was identified as having antibacterial activity in vitro against E. coli. None of the peptides had antibacterial activity against *B. cereus*. All synthesised peptides had ACE-1 inhibitory activities with values ranging from 81.68% and 76.78% ACE-1 inhibition for peptides VYNKFPYTTQ and NKFPYTTQ to 35.81% and 25.73% ACE-1 inhibition for peptides KGGGGGANGGRL and VVGAVGAAD respectively when assayed at a concentration of 1 mg/mL compared to the positive control Captopril© assayed at the same concentration with a reported ACE-1 IC_50_ value of 0.025 µM (Figure 3, *n* = 3). Interestingly, peptides NKFPYTTQ and VYNKFPYTTQ displayed the greatest ACE-1 inhibitory percentage values despite having Peptide Ranker scores of 0.19 and 0.18 respectively. The ACE-1 IC_50_ values for peptide VYNKFPYTTQ and NKFPYTTQ were determined as 347 µM (0.62 mg/mL) and 0.892 µM (0.592 mg/mL), respectively. ACE-1 IC_50_ values were not determined for the other peptides as initial percentage inhibition values were less than 1 mg/mL when assayed. Peptides also displayed COX-1 and COX-2 inhibitory activities however inhibition observed was less than 50% for all peptides screened against both cyclooxygenase enzymes. Peptide YANDLLCMPI inhibited COX-1 by 34.5% and COX-2 by 47.65% when assayed at a concentration of 1 mg/mL relative to Resveratrol (positive control) assayed at the same concentration, which inhibited both enzymes by 78.41 (±2.92) and 70.41%, respectively. The structure of the peptides are shown in Figure 4.

## 4. Discussion

Protein was extracted from *Nannochloropsis* sp. biomass using the ammonium sulphate salt out method as described previously [22,31] followed by enzymatic hydrolysis treatment with Xylanase enzyme from *Aspergillus oryzae*. In the course of the 32 years since the ammonium sulphate salt out method was first developed and applied to seaweeds, new extraction techniques were developed and were reviewed previously [45,46,47]. We used the method of Fleurence and colleagues [31] in combination with a novel enzyme treatment as seaweed protein extraction methods developed in recent years are not sufficient to obtain a satisfactory protein extract yield beyond what is achievable with the ammonium sulphate precipitation method. Angell [48] highlighted the need to improve protein extraction methods for seaweed if the paradigm of using seaweeds as an alternative protein crop is to be delivered. Work carried out by the Angell group found that proteins from the green seaweed *U. ohnoi* were most effectively isolated by adopting methodologies for terrestrial leaves but that the best protein isolate yields were lower than other studies for seaweeds [48].

According to a recent publication by de Amorin and colleagues [49], the enzymes Thermolysin and Bromelain are the most commonly used enzymes to generate bioactive peptides from biomass including algae [49]. We employed the enzyme Xylanase in our work as some xylanases are capable of degrading the crystalline microfibrils of 1,3-xylan that reinforce the cell walls of some algae [50]. *Nannochloropsis* species possess a thick and multilayered cell wall composed of polysaccharides and algaenan [51]. The inner part of the cell wall is made of cellulose and glucose and amino acids represent an integral cell wall constituent. Sugars including rhamnose, mannose, ribose, xylose, fucose and galactose are also found here. Use of xylanase enzymes was previously shown to enhance the enzymatic hydrolysis of lignocellulosic substrates [52]. In addition, xylanases are used in bread making to increase volume of the bread and contribute to improved bread quality, faster baking times and increased yields [25]. Wheat flour is treated with xylanase to break down hemicellulose, increasing the binding of water to the flour, allowing it to become softer and more elastic [53]. The protein content of extracted protein from *Nannochloropsis* sp. was 26.16% compared to a protein content of 27.95% in the whole microalga. The ash content of the generated protein extract was 27.78% compared to an ash content of 23.18 for the whole microalga. Yields compared favourably with yields obtained in previous studies from *Nannochloropsis* sp. (Table 1). 10-kDa MWCO filtration was used to enrich the protein extract for peptides prior to MS analysis. Bioactive peptides are usually between 2–30 amino acids in length (usually less than 10 kDa in size) and permeates less than 10 kDa have been found to be more effective antioxidants and antihypertensive agents previously [54].

Ninty five peptides were identified using MS and eight of these were synthesised. ACE-1 inhibitory peptides were identified following MS analysis of the 10 kDa MWCO enriched permeate. The peptide amino acid sequences correspond to NKFPYTTQ, VYNKFPYTTQ, LVGADAHALGVICS and VVGAVGAADLL. Identified peptides were not found in databases including the BIOPEP-UWM™ database of bioactive peptides (https://biochemia.uwm.edu.pl/biopep-uwm/ accessed on 2 August 2022) and PepBank (http://pepbank.mgh.harvard.edu, accessed on 2 August 2022). Analysis of these peptide sequences using PeptideRanker (http://bioware.ucd.ie/~compass/biowareweb/-accessed on 25 July 2022) found that they were unlikely to be bioactive as they all had scores less than 0.39. PeptideRanker was trained at a threshold of 0.5 i.e., any peptide predicted over a 0.5 threshold is labelled as bioactive. However, this database was designed for antimicrobial peptide predictions initially [39]. ACE-1 inhibitory peptides longer than four amino acids in length are bioactive if they have a bulky side chain aromatic amino acid at the C-terminal end of the peptide. NKFPYTTQ and VYNKFPYTTQ identified in this study have the polar, uncharged amino acids threonine and glutamine at their C-terminal end in the penultimate and ultimate positions [55]. Tyrosine (Y) is found in both peptides–this is an aromatic amino acid known to play a role in ACE-1 inhibition. In many potent ACE-1 inhibitor peptide sequences, tyrosine, phenylalanine, and tryptophan residues are also present at the C-terminus particularly for the di-and tripeptide inhibitors [56]. Indeed, the peptide TTQ which shares three amino acids with both NKFPYTTQ and VYNKFPYTTO was previously identified as an ACE-1 inhibitor isolated from beans previously [43]. Samarakoon and colleagues previously identified two ACE-1 inhibitory peptides from *Nannochloropsis*. sp. biomass using pepsin hydrolysis followed by ultrafiltration (UF) [8]. The peptides identified were GMNNLTP and LEQ with ACE-1 IC_50_ values of 123 and 173 µM respectively and were isolated using Sephadex G-25 gel filtration. The IC_50_ value identified for the permeate fraction in this study of 0.37 mg/mL (370 µg/mL) compares favourably to other protein permeate and peptide ACE-1 inhibitory values identified previously (Table 1). For example, a water soluble hydrolysate of *Chlorella vulgaris* was prepared previously using acid and cellulase enzymes and an ACE-1 IC_50_ value of 286 µg protein/mL determined [57].

The COX-1, COX-2 and antibacterial activities observed.

Following a simulated in silico digestion using PeptideCutter, several peptides previously identified as being ACE-1 inhibitors or having additional bioactivities including CAMKII inhibition and anti-thrombotic activity were identified [41,42,43,44,45]. This suggests that following digestion, the identified peptides could act beneficially to reduce the risk of thrombus formation and act through inhibition of CAMKII to improve further cardiovascular health [58]. Future work will involve synthesis of identified peptides and screening to confirm in vitro the ability of these peptides to inhibit CAMKII. This work has identified several novel bioactive peptides with activities ranging from ACE-1 inhibition to antibacterial. The peptide sequences are novel and are not in the literature or relevant databases. They offer potential for use in human functional foods or feeds for companion animals to maintain heart health and prevent infection. However, animal trials including the permeate extracts or synthesized peptides are required in the future.

## 5. Conclusions

Use of traditional ammonium sulphate precipitation combined with xylanase enzyme treatment and 10 kDa MWCO filtration was used to generate an ACE-1 inhibitory permeate fraction with an ACE-1 IC_50_ value of 370 µg/mL. 95 novel peptide sequences previously not published in the literature or found in bioactivity peptide databases like BIOPEP-UWM or PepBank were identified using MS, and in silico work identified the potential bioactivity of these peptides and also their ability to act as pro-peptides prior to simulated gastrointestinal digestion. Based on in silico analysis, eight peptides were chemically synthesised and tested in vitro for their antibacterial, ACE-1 inhibitory and COX inhibitory activities. The peptide NKFPYTTQ was identified as the most potent ACE-1 inhibitory peptide compared to the positive control Captopril©. This peptide has a Peptide Ranker score of 0.19 and was not found to be antibacterial in vitro. However, the sequence of the peptide, with a Q amino acid at the C-terminal end suggests that it was an ACE-1 inhibitor. This was confirmed in vitro. Peptide AGDVGFDPLGF was identified as an antimicrobial peptide as it produced a zone of inhibition against the pathogen *E. coli* but not *B. cereus* when assessed at a concentration of 1 mg/mL compared to controls. This peptide has a net charge of −2 and had a Peptide Ranker score of 0.89 indicating that it was likely antibacterial in nature but it is novel following a search of the literature and BIOPEP database. This peptide is not as potent as previously identified antibacterial peptides such as Sarconesin II (IC_50_ value of 1.90 µM–peptide ID 9546 BIOPEP) [59]. Few of the peptides listed in BIOPEP have reported EC_50_ or IC_50_ values for antibacterial activities. In vitro results confirm that it had activity compared to controls Ampicillin and Cecropin P1 against *E. coli*. In silico, digestion of identified and synthesised peptides identified the potential of these peptides to act as ACE-1 inhibitory peptides, antimicrobial, anti-thrombotic and CAMKII inhibitors [23]. The generated *Nannochloropsis oculata* permeate has potential to be used as a source of protein with added nutritional and functional food benefits due to the observed protein content of the permeate and in vitro and in silico results regarding bioactivities. Future work will include synthesis of the digested peptides and screening to confirm anti-thrombotic and CAMKII inhibitory activities.

## Figures and Tables

**Table 1:** Proximate compositional analysis of the whole *Nannochloropsis* sp. biomass used for protein and peptide generation in this study and extracted protein from the same biomass, compared to compositional analysis carried out for *Nannochloropsis* sp. previously.

Species	Proteins [% DW]	Lipids [% DW]	Carbohydrates [% DW]	Other [% DW]	References
*Nannochloropsis* sp. biomass (this study) *Nannochloropsis* sp. protein extract (this study)	27.95 ± 1.42 26.16 + 0.4	1.25 ± 0.39 0.38 ± 0.3	n/d n/d	Ash 23.18 ± 2.59; Moisture 6.37 ± 0.75 27.78 ± 0.13	This study This study
*Nannochloropsis gaditana*	46.2 ± 0.3	31.9 ± 0.6	15.8 ± 0.4	Ash 6.4 ± 0.0	Verspreet et al., 2021 [6]
*Nannochloropsis oculata*	30.4 ± 1.8	20.5 ± 1.2	37.1 ± 1.7	Mineral, fiber, etc. 11.1 ± 0.8	Qian et al., 2013 [34]
*N. oculata*	31.0 ± 0.0	1.3 ± 0.0	17.8 ± 0.1	Ash 32.9 ± 0.0 Fiber 4.4 ± 0.0	Samarakoon et al., 2013 [35]
*N. oculata*	30.5	8.0	19.6	Ash 30.6	Sanjeewa et al., 2016 [36]
*N. gaditana*	28.0	18.4	45.0	-	Nacer et al., 2020 [10]
*Nannochloropsis* sp.	31.7 ± 0.1	15.0 ± 0.1	9.0 ± 0.4	Ash 27.3 ± 0.2	Fithriani et al., 2020 [37]

**Table 2:** Bioactive peptides identified from *Nannochloropsis* sp. protein xylanase treated permeate fraction and associated bioactivities of peptide fragments derived from the same peptides following simulated gastrointestinal digestion with Pepsin and Trypsin enzymes.

Parent Protein Name & UniProt Accession Number	Peptide Single Amino Acid Sequence	Peptide Ranker Value ^1^	Novelty (Found in Database ^1,2^)	Observed Bioactivity In Vitro	Simulated Digestion Using PeptideCutter ^3^/Peptide Digestion Fragments	Associated Predicted Bioactivities of Peptide Fragments Resulting from Simulated GI Digestion	References
Chlorophyll a-b binding protein, chloroplastic OS = *Porphyridium purpureum* OX = 35,688 GN = FVE85_6435 PE = 4 SV = 1; Chlorophyll a-b binding protein 1B-21, chloroplastic OS = *Porphyridium purpureum* OX = 35,688 GN = FVE85_3955 PE = 4 SV = 1	AGDVGFDPLGF	0.89	Novel	ACE-1 inhibitory, Antibacterial activity against *E. coli* at a concentration of 1 mg/mL	Chymotrypsin–position 6 and 9. Peptide fragments AGDVGF, DPL and GF	No predicted bioactivities associated with any peptide fragments reported in BIOPEP	[26]
Mannose-6-phosphate isomerase OS = Vibrio sp. SM1977 OX = 2,662,262 GN = manA PE = 3 SV = 1; Uncharacterized protein OS = Porphyridium purpureum OX = 35,688 GN = FVE85_9013 PE = 4 SV = 1; REVERSED p-aminobenzoic acid synthase OS = Ectocarpus siliculosus OX = 2880 GN = PABS PE = 4 SV = 1; REVERSED Uncharacterized protein OS = Gimesia algae OX = 2,527,971 GN = Pan161_23400 PE = 4 SV = 1; REVERSED NADH-ubiquinone oxidoreductase chain 4 OS = Guillardia theta OX = 55,529 GN = nad4 PE = 3 SV = 1; REVERSED NADH-ubiquinone oxidoreductase chain 4 OS = Chroomonas placoidea OX = 173,977 GN = nad4 PE = 3 SV = 1; REVERSED Transcriptional regulator OS = Formosa algae OX = 225,843 GN = BKP44_19185 PE = 4 SV = 1; REVERSED Deoxyguanosine kinase OS = Shewanella algae OX = 38,313 GN = BFS86_13800 PE = 4 SV = 1	GDVGLF	0.82	Novel	ACE-1	Pepsin position 4, 5, 6, Chymotrypsin position 5 and 6. Peptide fragments GDVG, L, F	No predicted bioactivities associated with peptide fragments reported in BIOPEP	[26]
Uncharacterized protein OS = Mesonia algae OX = 213,248 GN = LX95_02145 PE = 4 SV = 1	YANDLLCMPI	0.77	Novel	ACE-1; COX-1 and COX-2 inhibition	Pepsin 1, 4, 5, 6 and Chymotrypsin at position 4, 5 and 6. Peptide fragments Y, AND, L, L, CMPI	MPI corresponds to amino acid fragments 2–5 of the antithrombotic peptide DMPIQAFLLYQEPVLGPVR, AND has sequence similarity with peptides NDQF an antiviral peptide and NDPQF a bitterness suppressing peptide but no reported exact matches for AND	[26]
REVERSED SHR-BD domain-containing protein OS = *Ectocarpus siliculosus* OX = 2880 GN = Esi_0004_0132 PE = 4 SV = 1	KGGGSGANGGRL	0.72	Novel	ACE-1	Pepsin, Trypsin and Chymotrypsin cut the peptide at positions 1, 11 and 12. Resulting peptide fragments are K, GGGSGANGGR, L	GGGSGANGGR has sequence similarity in terms of the amino acids GR with peptides GGAAGGR a DPP-IV inhibitory peptide, TKHGGRINTL an antiviral peptide and the ACE inhibitory peptide FPVGRGL	[26]
Phycocyanin protein, accession number: tr|S5FXR4|S5FXR4_9CYAN	NKFPYTTQ	0.19	Novel	ACE-1 inhibition	Trypsin–position 2 Pepsin–position 3 Peptide fragment: NK, F, PYTTQ	NK–ACE-1 inhibitor found previously in Wakame seaweed; TTQ found as an ACE inhibitor previously isolated from bean (*Phaseolus vulgaris*)	[41,42]
Protein R-phycocyanin alpha subunit *Synechococcus* sp. (accession number: P27288 PHCA_SYNPW	VYNKFPYTTQ	0.18	Novel	ACE-1 inhibition	Trypsin–position 4 Pepsin–1, 2, 5 Resulting peptide fragments: V, Y, NK, F, PYTTQ	NK–ACE inhibitor found previously in Wakame seaweed; TTQ found as an ACE inhibitor previously isolated from bean (*Phaseolus vulgaris*)	[43]
Transcriptor initiation protein from *Porphyridium* sp. (accession number tr|A0A5J4YUD5|A0A5J4YUD5_PORPP	LVGADAHALGVICS	0.39	Novel	ACE-1 inhibition	Pepsin–positions 1 and 8 Resulting peptide fragments: L, VGADAHA, LGIVICS	AHA, also occurs at the C-terminal end of a peptide NGRAHA that has antithrombotic activity;	[44]
HEAT repeat-containing protein 1 OS = *Ectocarpus siliculosus* OX = 2880 GN = Esi_0125_0072 PE = 3 SV = 1 (accession number: tr|D7FJ23|D7FJ23_ECTSI)	VVGAVGAADLL	0.23	Novel	ACE-1 inhibition	Pepsin at positions 9, 10 and 11 Fragments: VVGAVGAAD, L, L	AAD found at C-terminal end of CAMKII inhibitor KKALRRQEAADAL	

^1^ https://biochemia.uwm.edu.pl/biopep-uwm/. ^2^ http://pepbank.mgh.harvard.edu. ^3^ https://web.expasy.org/peptide_cutter/ (accessed on 26 July 2022).

## Data Availability

Data is available from the corresponding author concerning this study.
